# Abstract of Proceedings of the Buffalo Medical Association

**Published:** 1868-06

**Authors:** T. M. Johnson

**Affiliations:** Sec’y.


BUFFALO

Medical and Surgical Journal.

VOL. VII. JUNE, 1868. No. 11.

### Original Communications.

ART. I.—*Abstract of Proceedings of the Buffalo Medical Association.*

TUESDAY EVENING, May 5th, 1868.

The meeting was called to order by the President, Dr. J. R. Lothrop. Members present—Drs. Lothrop, Congar, Shaw, Smith, Abbott, Potter, Trowbridge, Wetmore, Samo and Johnson.

Report of the minutes of the last meeting accepted. After which Dr. J. R. Lothrop made the following remarks:

This occasion does not call for, nor does my inclination lead me to much speaking, at this time. Your time can be better employed and suggestions had better follow than precede service. Especially would they be inopportune now, when we hope *soon* to receive them more practical, fit, and wise from our retiring President. But I will say a few words, more to show that I do not slight the occasion, than from any feeling that they may profit you in any degree.

Entering upon the duties of the office to which you have chosen me, I desire to express my sincere thanks for this proof of your generosity and confidence. That you have deemed me fit for it ought to be enough to lead me to work to the extent of my ability to promote the great object for which this Association was formed. I hope I may be able to do this, but I am impressed with a becoming distrust when I remember the highly useful and honorable history of this Association. Many things have been said here which have been widely read and much thought of. This Society has had a broad utterance, and may with just pride speak of its work and claim its share of honor. It is entitled to receive this in equal measure with any kindred association in the land, for none has been more earnest or progressive. But not alone for what it has done should we cherish it; nor yet because what is said here may be read by many and exert a wide influence. We should remember that the best thing it may give us, is self-improvement. We should prize it as a source of instruction to ourselves.

The prime object of our meetings here is to derive benefit and instruction from our related experience and the discussion and remarks which grow out of it. The discussions are not for the purpose of setting up or maintaining mere pride of opinion, but to elicit truth. And while for that object difference of opinion is more or less to be looked for, all opinions should meet with just consideration. Nothing is more difficult than rightly to interpret experience. It may convey to different minds equally candid, equally earnest to know what is actually true, lessons very unlike, if not indeed directly opposite. The fallacies of experience are familiar enough to all of us, and while we must not allow ourselves to be drawn into skepticism on the one hand, or exclusiveness on the other; nay, while we must not give these fallacies of experience too cordial reception, and thereby fall into the bad habit of looking at experience rather for its fallacies than its truths; we may and ought to question hasty conclusions and opinions, although they claim to be founded upon experience. Careful observation alone makes experience valuable, and its deductions sound, and consequently no contributions are more interesting or instructive than cases. Even the most common cases, well stated, are instructive always, and especially a well-arranged collection of such. Every member can give his share of these, the younger as well as the older, and there is no necessity for waiting for novelties or strange things, while the ordinary experience is always repeating itself and always teaching something new. Time, and better methods of study, are every day proving to us that what seems best known is yet capable of yielding to us new truths, and there are but few things of which it can be said they are as well known as they can be. None are more ready to admit this than medical men, and hence the immense stimulus which directs all their studies. Every young man may feel that to be thoroughly progressive there is no need that he should travel into new regions, for by faithfully going over the old ground, he can add to our knowledge, and if he fails to make himself a discoverer, he can furnish those well-considered and conscientious labors from which discoveries arise.

It will not, I trust, be out of place to suggest a more frequent relation of well-observed cases, which will profit him who observes and relates, and him who hears. But there is no need that I should seem to urge you to that effort for mutual improvement—that effort of which you, the present members, and those now no longer associated with us, have given such conspicuous proof. Each should remember that, whatever his gifts or opportunties, he deserves well, yes the *best*, who is true to the best that there is in him. And, I may say, deserves better than he who is willing to give us less than the best of which he is capable.

Dr. M. G. Potter read the following paper, for which he received a vote of thanks:

Your attention is this evening invited to a brief review of the arguments already advanced, both for and against the theory which attributes to mercury the power of promoting the secretion of bile, and also to a new method of determining whether mercury possesses this power. Until within a few years no theory in medicine was more strongly corroborated by the concurrent belief, universal practice and experience of the medical world, than that mercury increases the biliary secretion. The general *malaise* and indefinite symptoms which were universally attributed to torpidity of the liver were relieved by it. Clay colored stools which were thought to denote absence of bile were rendered green or dark by the employment of mercury, and this green or dark color was attributed to the presence of bile. Indeed this change of color in the fæces was confidently relied upon by most practitioners, and it is still relied upon in the U. S. Dispensary, as a certain proof that mercury stimulates the liver and promotes the secretion of bile.

Another argument in support of this theory has been adduced from what is known of the action of mercury on the other glands of the body. It is believed that mercury is not a component part of the body, and that nature therefore strives to get rid of it, and that it is accordingly eliminated from the system, passing out through the glands. Mercury is eliminated through the bowels. This is shown, not by internal administration, for all know that a portion only of certain of the mercurial preparations is absorbed, the remainder passing into the condition of fæces. But when administered by inunction mercury has been detected in the alvine discharges. It probably gains entrance into the intestines through the liver in the bile, and through the pancreas in the pancreatic liquid. Certainly as it exists in the blood it is soluble in the bile and pancreatic liquid, and it has, I think, been detected in both these secretions. Mercury is also eliminated from the system through the kidneys. It can be found in the urine of persons taking it. Mercury likewise passes out of the body through the parotid glands in the saliva. It has been repeatedly detected in the saliva of persons while ptyalized; and the point worthy of special notice in this connection regarding the action of mercury on the parotid glands is, that coincident with the appearance of mercury in their secretions the quantity of saliva is perceptibly augmented. In its exit from the body through these glands mercury therefore excites them to increased secretion. As this effect is capable of ocular demonstration, none can be found to deny that it belongs especially to the drug under consideration.

But mercury quits the system through the kidneys and skin and pancreas, and their secretions are respectively increased by it. Mercury is therefore diuretic and diaphoretic, and by its action the pancreatic liquid is augmented. Few can be found to deny these effects of mercury. And it is claimed that mercury increases the flow of bile precisely as it increases the flow of saliva, and of urine, and of perspiration, and of pancreatic liquid. In other words that mercury is eliminated from the system through the liver in the bile, and the rule of other eliminatives is not departed from, the biliary secretion being augmented.

Opposed to this theory, however, is the belief at present entertained by many that mercury not only does not increase the biliary secretion, but that this secretion is, if anything, diminished by its employment. It is affirmed that the green color of calomel stools is due not to bile, but to the sub-sulphide of mercury, precisely as the black color of stools following the administration of iron is due to the sub-sulphide of iron. It is likewise affirmed that the intestinal contents are not of this green or dark color before passing the illio-cæcal valve; and hence that this color is so far independent of bile that it is acquired in the large intestine, especially the colon. And it is still further affirmed that the dark colored fæces in the colon yield upon analysis less bile than the lighter colored contents of the small intestine.

And furthermore, with a view to settling this question, experiments have been made upon dogs by five different and competent observers, these experiments consisting in the establishment of a biliary fistula, the closure of the common bile duct, and then determining the quantity of bile secreted without mercury, and comparing this with the quantity secreted under its influence; and these experiments indicate pretty clearly that mercury does not increase the amount either of fluid bile, or bile solids in the dog, after a biliary fistula has been established. But there is possibly a difference in the action of mercury on dogs and on men, and certainly these experiments cannot be said to prove that mercury does not increase the biliary secretion in men. And, it seems to us, that those who deny the cholagogue action of mercury have most signally failed in substantiating their denial, so far at least as that denial is based on their analysis of the fæces. For the position of those claiming the biliary secretion to be increased by mercury, is not in the least invalidated by a proof that the dark colored fæces in the colon contain less bile than the lighter colored contents of the small intestine. Nor is this position shaken by a proof that the green color of calomel stools following clay colored stools does not depend upon bile. Nor is the position made untenable even by a proof that bile is absent from the fæces after calomel has been administered. These statements, if true, (and I have no doubt they are,) simply prove that the old argument, from change of color in the fæces to prove the cholagogue action of mercury, is invalid. But a conclusion is not always false because one or both the premises which have been associated to sustain it are proved false. It may be intrinsically true, and still the argument advanced to prove it may be most unfortunate. And this, it seems to me, may be the case with the premises from which the proof of the cholagogue action of mercury has hitherto been drawn. They may be false, but to prove the action itself false requires additional investigation. And why do these statements not disprove the cholagogue action of mercury? Those who claim this for them have, it seems to us, taken too superficial a view of this matter. For what becomes of the bile in health after it passes into the intestines? Were it simply excrementious, were it secreted, simply to be eliminated from the system unchanged with the fæces, these statements would be conclusive proof that mercury does not increase the biliary secretion.—Unfortunately for the objectors, however, this is not the truth with reference to the behavior of bile. For, says Dalton, p. 192, "The results thus obtained (from experiments which he had performed to determine what becomes of the bile) show that under ordinary circumstances the bile, which is quite abundant in the duodenum and upper part of the small intestine diminishes in quantity from above downward, and is *not to be found* in the large intestine. The entire quantity of intestinal contents also diminishes and their consistency increases, and at the same time their color changes from a light yellow to a dark bronze or blackish green, which color is always strongly pronounced in the last quarter of the small intestine." The conclusion further on is reached that the "biliary matters disappear in their passage through the intestine." And these views are confirmed by the researches of Prof. A. Flint, jr., vol. 1, p. 34, "In our examination of human fæces we have failed by Pettenkoffer's test to detect the slightest trace of the biliary salts." And Wehsarg has shown that the color of the fæces varies with the diet; a mixed diet producing a yellowish brown color; a milk diet, a more decided yellow, while an exclusively flesh diet gives color much darker than either. Therefore examinations of the fæces for bile are not to be relied on to determine whether the secretion of the liver is diminished, for if bile cannot be detected in the fæces when its amount is normal, it is most improbable that it can be detected when its amount is less than normal. Nor can these examinations be relied on to determine whether the biliary secretion is increased; for if increased, we have no reason to believe that the excess would not do as the normal amount does, namely: "*disappear* in its passage through the intestines." In other words, there is no reason to believe that this excess would appear in the fæces. But suppose that this excess does appear in the fæces and is recognized by Pettenkoffer's test, what would be the logical deduction from this state of things? If that there was an excess of secretion the fact would tend at least to prove the cholagogue action of mercury. But clearly, this conclusion does not necessarily follow, for the presence of bile in the fæces could result from such a derangement of the intestines as would prevent the natural process by which the bile disappears in its passage through them.

This problem is, therefore, most difficult of solution, for its solution cannot depend on the result of fæcal analysis for bile, nor can it be aided by those results; nor can mere inspection of the fæces throw any light on this matter, for there is certainly as much reason to believe that the green color of calomel stools is independent of bile as to believe that this color is caused by bile. We must therefore seek our answer to this question in a different way. And it seems to me that it is possible to determine accurately whether the biliary secretion is increased by mercury. You are aware that there is in the bile an excrementious principle, cholesterine, and that this substance, either naturally or by processes employed for its extraction, is converted into stercorine, being found as such in the fæces. Now there is a definite relation between the amount of stercorine discharged and the amount of bile secreted. If, therefore, we determine the amount of stercorine discharged before the administration of mercury, and compare this with the amount discharged after mercury has been given, we can determine the effect of mercury on the biliary secretion, so far at least, as increase or diminution is concerned. I am not aware that examinations of the fæces for stercorine have been made with a view of determining whether the flow of bile is promoted by mercury. But if the physiologists have taught us correctly concerning this substance these examinations can scarcely do less than settle the question.

Dr. Lothrop said that he had listened to the paper with much interest. It seemed very clear that the color of the discharges from the intestines could not be relied upon to prove an excess or deficiency in the amount of bile secreted. He had touched upon this matter in a paper read before the Society some time since, but he was pleased with the fuller and clearer statement of it in the paper just read. Moreover it did not appear that the quantity of stercorine, which escaped from the body, gave any absolute measure of the quantity of bile. Stercorine is the representative of the excrementitious portion of the bile alone. The bile is a double fluid, a secretion and an excretion, and there is no reason to believe that the two elements exist in a definite proportion. There might be an excess of one or the other, at any rate the proportion may be, and probably is variable, hence the amount of one for a given time would not enable us to estimate certainly the amount of the other.

Dr. Congar said he had long been convinced that mercury stimulated the flow of the bile. It occurs to me that the test mentioned by Dr. Potter would apply to all the cholagogue medicines.

Dr. Trowbridge said that he was educated to believe that a certain change in the condition of the stools was positive evidence of the action of mercury. In cases of cholera my father and most, if not all, the physicians of his day, considered calomel and opium the sheet-anchor, and looked for a change in the color of the stools as an indication of action of mercury on the liver and the safety of their patients.

Dr. Lothrop said that the change in the appearance of the discharges in cholera was undoubtedly a favorable symptom. It was not, however, to be taken as an indication that the calomel had acted favorably. There was no good reason for believing that calomel had any agency in causing it. The re-appearance of the other excretions was equally favorable. Of the fact there could be no doubt, but he could not admit that it had any clear connection with the calomel made use of.

Dr. Congar remarked that the experiments of Dr. Little of London, proved that the injection of salines into the veins caused a similar change in the stools to that caused by the administration of mercury.

Dr. Trowbridge replied that, the statement of Dr. Congar is not proof that mercury may not change the character of fæcal matter. I must continue to believe that mercury has its action upon the liver, stimulating it to increased action, one of the results of which is a change in the fæcal matter.

Dr. Smith said he had been interested in the paper read. The author has mentioned a good method for detecting bile in the fæces. I have always found a change in the fæcal matter in diarrhœa, cholera infantum, etc., a favorable symptom. Have often remarked the change after giving calomel. Believe it will be found that calomel has a decided action upon the liver.

Dr. Johnson reported the following case: Was called last week to attend a man about forty-five years of age, whom I found in a nearly insensible condition, could articulate but one or two words; pulse quite rapid and feeble; the skin and conjunctiva were very much jaundiced. He had considerable cough, and on examination found pneumonia of the left lung. His condition was such as to convince me that he could live but a few hours at most. Ordered stimulants to be given freely, which, however, had but little effect upon him. He died eight hours after my first visit.

*Post-mortem* examination showed a pneumonial condition of nearly the whole of the left lung and considerable adhesion of the left pleura. The liver was enlarged to about once and a half its normal size, and presented all the usual appearances of cirrhosis, except the external lobulated or hobnail appearance. On making incisions into it the tissue was found firm and hard, and thickly studded with lobules of yellow cheesy matter. The spleen, which I here present for your inspection, weighs twenty-six ounces, and is therefore nearly four times the normal size of this organ. On bi-secting we find the cut surface thickly studdied with the yellow cheesy matter like that found in the liver; the lobules being rather more prominent than they were in the liver. We have comparatively often seen cirrhosis confined to the liver, but have never seen it involve both the liver and spleen to this extent. The man was an entire stranger in this city, and being unable to converse, his former history could not be obtained. All the indications in the case warrant the belief that he was an intemperate man, and that the disease of the liver and spleen was caused by an intemperate use of alcoholic stimulus.

Dr. Lothrop thought that the enlargement was due to the deposition of an anomalous fibrous substance in the tissues. He supposed the liver was affected in a corresponding manner, and that had life been prolonged gradual diminution in size would have occurred, while the induration would have become greater. In other words, it was the enlargement which often, if not always, takes place in that disease known as granular liver. The increase in size in the spleen he supposed had no necessary connection with that of the liver; though, occurring together, they probably arose from the same cause.

No special disease was reported as prevailing.

T. M. Johnson, Sec'y.

